# Design, Development, and Clinical Validation of a Novel Kit for Cell-Free DNA Extraction

**DOI:** 10.3390/diagnostics15151897

**Published:** 2025-07-29

**Authors:** Ekin Çelik, Hande Güner, Gizem Kayalı, Haktan Bagis Erdem, Taha Bahsi, Hasan Huseyin Kazan

**Affiliations:** 1 Department of Medical Biology, Faculty of Medicine, Kırşehir Ahi Evran University, Kırşehir 40100, Türkiye; 2 ZipPrime Biotechnology Co., Eskişehir 26190, Türkiye; hguner@zipprime.com (H.G.); gkayali@zipprime.com (G.K.); 3 Department of Medical Genetics, Ankara Etlik City Hospital, Ankara 06170, Türkiye; haktanbagis@gmail.com; 4 Department of Medical Genetics, Ankara Dr. Abdurrahman Yurtaslan Oncology Training and Research Hospital, University of Health Sciences, Ankara 06200, Türkiye; tahabahsi@yahoo.com; 5 Department of Medical Biology, Gulhane Faculty of Health Sciences, University of Health Sciences, Ankara 06010, Türkiye; hasanhuseyinkazan@gmail.com

**Keywords:** cell-free DNA, cfDNA extraction, magnetic bead-based isolation, liquid biopsy, pre-analytical optimization

## Abstract

**Background:** Cell-free DNA (cfDNA) has become a cornerstone of liquid biopsy applications, offering promise for early disease detection and monitoring. However, its widespread clinical adoption is limited by variability in pre-analytical processing, especially during isolation. Current extraction methods face challenges in yield, purity, and reproducibility. **Methods:** We developed and optimized SafeCAP 2.0, a novel magnetic bead-based cfDNA extraction kit, focusing on efficient recovery, minimal genomic DNA contamination, and PCR compatibility. Optimization involved systematic evaluation of magnetic bead chemistry, buffer composition, and reagent volumes. Performance was benchmarked against a commercial reference kit (Apostle MiniMax) using spiked oligonucleotides and plasma from patients with stage IV NSCLC. **Results:** The optimized protocol demonstrated superior recovery with a limit of detection (LoD) as low as 0.3 pg/µL and a limit of quantification (LoQ) of 1 pg/μL with no detectable PCR inhibition. In comparative studies, SafeCAP 2.0 showed equivalent or improved performance over the commercial kit. Clinical validation using 47 patient plasma samples confirmed robust cfDNA recovery and fragment integrity. **Conclusions:** SafeCAP 2.0 offers a cost-effective, high-performance solution for cfDNA extraction in both research and clinical workflows. Its design and validation address key pre-analytical barriers, supporting integration into routine diagnostics and precision medicine platforms.

## 1. Introduction

Extracellular DNA (cell-free DNA; cfDNA) is the term used to describe DNA that is free in body fluids and can be used as a marker for the identification and monitoring of various physiological and pathophysiological conditions [1]. cfDNA analysis has shown great potential in the early diagnosis of fatal diseases such as cancer, in the monitoring of inflammatory processes, and in the follow-up of clinical procedures like organ transplantation [2,3]. However, the lack of standardization of pre-analytical procedures for cfDNA analysis limits its widespread use in clinical applications [4].

cfDNA in plasma, typically around 166 bp in length, is considered an ideal biomarker for disease prevention, diagnosis, treatment, and prognosis monitoring. This is due to its stability in body fluids rich genetic and epigenetic content, and the non-invasive nature of its collection [5,6,7,8,9,10]. In addition to cancer prognosis and monitoring, one of the earliest and most successful applications of cfDNA is in non-invasive prenatal testing (NIPT). NIPT detects fetal DNA circulating in maternal blood, allowing for the highly sensitive identification of genetic abnormalities [11].

Despite these advantages, research on cfDNA remains in its early stages, largely due to the complexity of its analysis, which involves multiple steps such as sample collection, processing, extraction, quality control, and long-term storage [12,13,14,15,16]. Additionally, challenges related to sample collection, prevention of short fragment loss, and the validation of various measurement methods further compromise the clinical reliability of cfDNA analysis by complicating comparisons and hindering reproducibility [17,18,19,20,21]. Moreover, the accuracy and reliability of cfDNA results are significantly affected by the methods of sampling and storage conditions, and transportation processes [22,23,24].

Proper handling and storage, coupled with efficient extraction of cfDNA whose characteristics are susceptible to alteration by biological and environmental variables are essential for ensuring the accuracy and reliability of analyses. However, given that cfDNA is typically present in low concentrations and is highly fragmented, the selection of an appropriate extraction method is of paramount importance [25]. Current cfDNA extraction techniques can broadly be classified as traditional liquid- or solid-phase-based isolation methods, improved isolation techniques employing advanced silica membranes or magnetic beads, and emerging technologies based on microfluidic systems or nanotechnology [26]. When selecting an extraction protocol, critical factors such as DNA yield, purity, reproducibility, and cost must be considered. Furthermore, specialized cfDNA extraction kits are commercially available for both manual and automated protocols, with automated extraction systems demonstrating a lower margin of error and superior reproducibility compared to their manual counterparts [27]. However, development of such isolation systems is still required to eliminate possible problems such as cost and labor and to increase stability and sensitivity [28]. To address these limitations, a magnetic bead-based approach was selected due to its compatibility with automation, scalability, and high surface area for nucleic acid binding. Compared to column-based or precipitation techniques, magnetic beads offer more efficient recovery of fragmented cfDNA, reduced hands-on time, and improved reproducibility—making them ideal for both research and clinical applications.

The aim of this study was to optimize and validate a magnetic bead-based cfDNA isolation kit, SafeCAP 2.0 Cell-Free DNA Extraction and Capturing Kit. The kit components were determined and optimized using spiked oligonucleotide fragments. The isolated cfDNAs were characterized by fluorometric and microelectrophoretic measurements, and the isolation performances were compared with a commercially available kit. For clinical validations, liquid biopsy samples were obtained from stage IV non-small cell lung cancer (NSCLC) patients, and cfDNA isolation was performed using the SafeCAP 2.0 Cell-Free DNA Extraction and Capturing Kit, and isolated DNAs were characterized by microelectrophoretic measurements. The overall results underlined a new cfDNA isolation kit ready to be used in the clinics.

## 2. Materials and Methods

### 2.1. Kit Development and Optimization

As a routine approach, kit components included magnetic beads, lysis buffer, proteinase K, binding buffer, wash buffers, and elution buffer [29]. The volumes and concentrations of the components were optimized by spiking artificial double stranded (ds) oligonucleotides into the commercial human plasma (Sigma Aldrich, St. Louis, MO, USA) [30,31]. For spiking, ds oligonucleotides (with sizes of 40–70 bp; oligo2: 80–100 bp; oligo3: 70–90 bp; or their combination) whose sequences were randomly determined were added into 0.2 mL of human plasma samples and isolations were conducted using developed and already available commercial kits.

### 2.2. Optimization of Magnetic Beads

Magnetic beads used in the development of the SafeCAP 2.0 kit were synthesized in-house using a co-precipitation method. Briefly, magnetic nanoparticles were prepared by alkaline co-precipitation of Fe^2+^ and Fe^3+^ salts under inert conditions, followed by stabilization and surface functionalization with specific chemical groups (e.g., –COOH or –OH) depending on the experimental group. Particle size was modulated through control of surfactant concentration and reaction conditions.

Six different bead formulations (B1–B6) with varying side groups, solid contents, and size distributions were evaluated for cfDNA recovery performance (Table 1). Beads were characterized by dynamic light scattering (DLS) to assess hydrodynamic diameter and polydispersity index (PDI), and by scanning electron microscopy (SEM) for morphological verification.

A standardized protocol was applied for the assessment of the beads. Oligonucleotide-spiked plasma (up to 50 ng, varying amounts) was first centrifuged at 1600× *g* at 4 °C for 10 min. The supernatant was collected and subjected to a second round of centrifugation under the same conditions, after which the supernatant was collected again. Next, 30 μL of proteinase K (20 mg/mL) and 200 μL of the pre-developed lysis buffer formulation—comprising guanidine HCl, a nonionic surfactant, Tris base, and a capturing agent—were added. The mixture was incubated at 300 rpm and 60 °C for 15 min using a thermal shaker. Following incubation, 1 mL of the pre-developed binding buffer (containing guanidine salt, sodium sulfate, and 2-propanol) was added, along with varying volumes of magnetic beads (10–40 μL). Each sample was processed separately for each bead type and volume. The mixture was then incubated at 400 rpm at room temperature for 10 min. After incubation, the mixture was placed on a magnetic rack to separate the beads from the supernatant, which was subsequently discarded. To ensure purity, the beads were washed twice using 500 μL of wash buffer I (containing guanidine salt and ethanol) and wash buffer II (containing ethanol). Finally, 40 μL of elution buffer was added, and the DNA was eluted by shaking at 800 rpm at room temperature for 5 min. The DNA-containing supernatant was collected using a magnetic rack. The size and concentration of the isolated oligonucleotides were analyzed using the 2100 Bioanalyzer System (Agilent Inc., Santa Clara, CA, USA), following the supplier’s standard protocol. The isolation yields were quantified and graphically represented to assess overall efficiency.

### 2.3. Optimization of Other Kit Components

Beyond magnetic bead optimization, the concentrations and volumes of other key kit components—including lysis buffer, proteinase K, binding buffer, and wash buffers—were refined using the same systematic approach. The lysis buffer contains guanidinium HCl, Tris, and Triton X-100 to ensure efficient disruption of protein–DNA complexes. The binding buffer includes isopropanol and polyethylene glycol to promote DNA adherence to magnetic beads. Washing buffers consist of ethanol-based solutions (70–80%) with low salt concentration, and the elution buffer is composed of Tris-HCl. However, due to commercial considerations, the precise concentrations and volumes of individual ingredients have not been disclosed in this study.

### 2.4. Limit of Detection Ratio

The limit of detection (LoD) of the developed kit was examined using spiking and re-isolation approaches explained above. Basically, the concentrations of the spiked oligonucleotides ranged between 2.5 and 0.001 ng/μL, and re-isolations were performed.

### 2.5. Assessment of PCR Inhibition

To assess the potential inhibitory effects of the kit components on PCR, both standard PCR and real-time (RT) PCR were performed using a spiking and re-isolation strategy with artificial oligonucleotides. Oligonucleotide-specific primer pairs were designed, and conventional PCR was conducted using a Taq polymerase kit (Thermo Scientific, Waltham, MA, USA). The template DNA (re-isolated oligonucleotide) was tested at varying concentrations ranging from 1 ng/μL to 0.001 ng/μL, and the resulting PCR amplicons were qualitatively assessed using 2.5% agarose gel electrophoresis. Additionally, potential RT-PCR inhibition was investigated using a SYBR Green Mastermix (NEB, Ipswich, MA, USA). Oligonucleotide-specific primer pairs were designed for RT-PCR, and template DNA (re-isolated oligonucleotide) at different concentrations (1 ng/μL to 0.001 ng/μL) was analyzed via real-time PCR on the AriaMx Real-Time PCR System (Agilent Inc., Santa Clara, CA, USA). The amplification curves were manually reviewed and qualitatively analyzed to detect any inhibitory effects.

### 2.6. Comparison of Performances

The isolation efficiency of the developed kit was systematically evaluated in comparison to a commercially available reference kit [32,33]. Oligonucleotides were spiked into 1 mL of human plasma and subsequently re-isolated using the Apostle MiniMax High Efficiency Cell-Free DNA Isolation Kit (Apostle, San Jose, CA, USA). Briefly, 40 μL of proteinase K (20 mg/mL) and 100 μL of sample lysis buffer were added into 1 mL of plasma containing spiked oligonucleotide. Next, the mixture was incubated at 60 °C for 20 min. After incubation, the samples were mixed with 1.25 mL of lysis/binding buffer and 15 μL of magnetic nanoparticles. The mixture was incubated at 1200 rpm for 10 min. After that, the samples were taken onto a magnetic rack, and the beads were washed twice with wash buffers. Finally, DNA was eluted with 20 μL of elution buffer by incubating at 1400 rpm for 3 min. The re-isolated DNA was analyzed using the 2100 Bioanalyzer System (Agilent Inc., Santa Clara, CA, USA) to assess yield and quality. The resulting DNA concentrations were plotted and compared against those obtained using the developed kit, providing a comprehensive evaluation of the relative performance of both isolation methods.

### 2.7. Clinical Validations

#### 2.7.1. Ethics

This study was approved by the Ethics Committee of Dr. Abdurrahman Yurtaslan Ankara Oncology Training and Research Hospital, University of Health Sciences (Approval No: 2021-04/1054). All research was performed in accordance with relevant guidelines/regulations. Prior to participation, written and verbal informed consent was obtained from all patients.

#### 2.7.2. Participants

A total of 47 patients diagnosed with stage IV non-small cell lung cancer (NSCLC) were enrolled in the Department of Medical Oncology and referred to the Department of Medical Genetics at Dr. Abdurrahman Yurtaslan Ankara Oncology Training and Research Hospital for molecular diagnostics between 2021 and 2025. For each patient, 4 mL of peripheral blood was collected in Cell-Free DNA Blood Collection Tubes (Streck, La Vista, NE, USA). The collected samples were subsequently processed for cfDNA isolation using the SafeCAP 2.0 Cell-Free DNA Extraction and Capturing Kit. The extracted cfDNA was then analyzed and characterized using the 2100 Bioanalyzer System (Agilent Inc., Santa Clara, CA, USA) to assess its quality and integrity.

### 2.8. Statistical Analysis

All statistical analyses were performed using GraphPad Prism 9.0 (GraphPad Software, Boston, MA, USA). Data were expressed as mean ± standard deviation (SD) or median (interquartile range, IQR), depending on the normality of the distribution. The significance level was set at *p* < 0.05. Yield and purity measurements of cfDNA isolated using different magnetic beads were compared using one-way ANOVA followed by Tukey’s post hoc test for multiple comparisons. The coefficient of determination (R^2^) was used to evaluate the linearity of cfDNA recovery. The lowest concentration at which the kit reliably detected cfDNA (signal-to-noise ratio (SNR) ≥ 3) was defined as the limit of detection (LoD). The cfDNA yield and purity obtained using the SafeCAP 2.0 Kit and the Apostle MiniMax Kit were compared using independent *t*-tests as the results were normally distributed. Bland–Altman analysis was used to assess agreement between cfDNA yields from the two kits [34]. All analyses were performed at a 95% confidence level (CI), and statistical significance was considered at *p* < 0.05.

## 3. Results

### 3.1. Kit Development and Optimization

**Figure 1:**
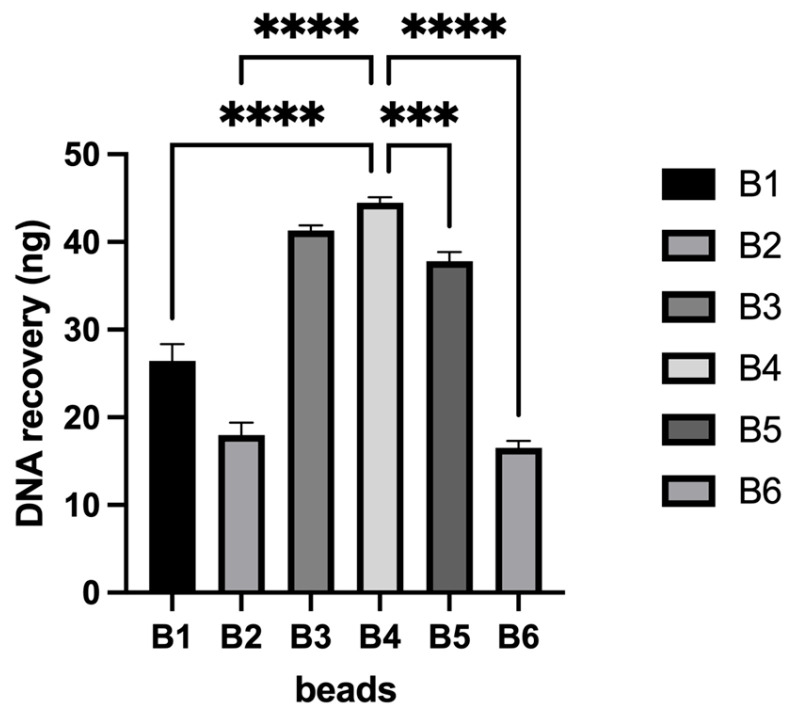
DNA yields obtained from the spiking and re-isolation process for the selection of optimal magnetic bead chemistry and size. Statistical significance was assessed as follows; *** *p* < 0.001, **** *p* < 0.0001.

**Figure A1:**
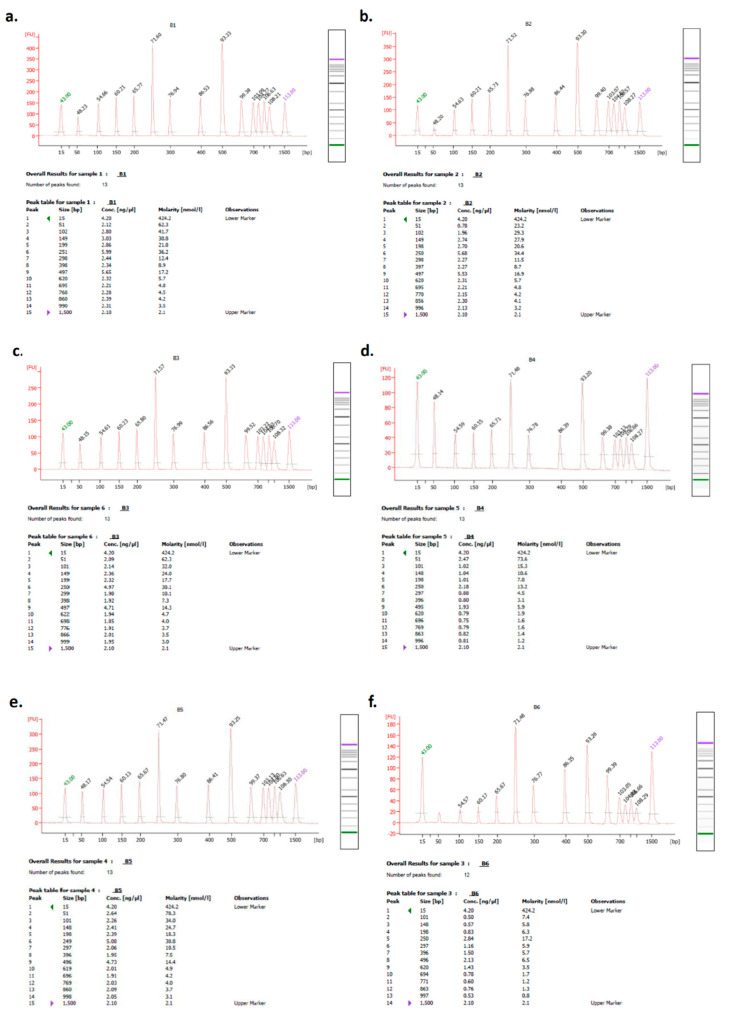
Microelectrophoretic measurements after spiking–re-isolation process for selection of the optimal magnetic beads. (**a**–**f**) B1–B6 bead chemistry.

The selection of magnetic bead chemistry was performed using an oligonucleotide spiking and re-isolation approach in human plasma as described above. Based on the experimental results, magnetic beads functionalized with -OH side groups and sized between 100 and 250 nm exhibited the highest efficiency in recovering spiked oligonucleotides (50 ng), yielding optimal DNA recovery rates (Figure 1 and Figure A1).

DNA recovery efficiency varied significantly among the different bead types (B1–B6), as shown in Figure 1. Bead types B3, B4, and B5 exhibited the highest DNA recovery, with mean values over 40 ng/µL, while B1, B2, and B6 demonstrated significantly lower recovery levels. Notably, B4 achieved the highest mean recovery, indicating superior binding and elution efficiency compared to the other bead types.

A one-way ANOVA revealed statistically significant differences among the groups (*p* < 0.05), followed by post-hoc pairwise comparisons confirming that B3 and B4 recovered significantly more DNA than the others (*p* < 0.01). There was no statistically significant difference in DNA recovery yield between B3 and B4 beads. Given their comparable performance, B4 beads were selected for further experiments based on additional qualitative factors, including stable dispersion in buffer, minimal aggregation, compatibility with the buffer system, and efficient magnetic separation, which collectively supported their ease of handling and reproducibility.

**Figure 2:**
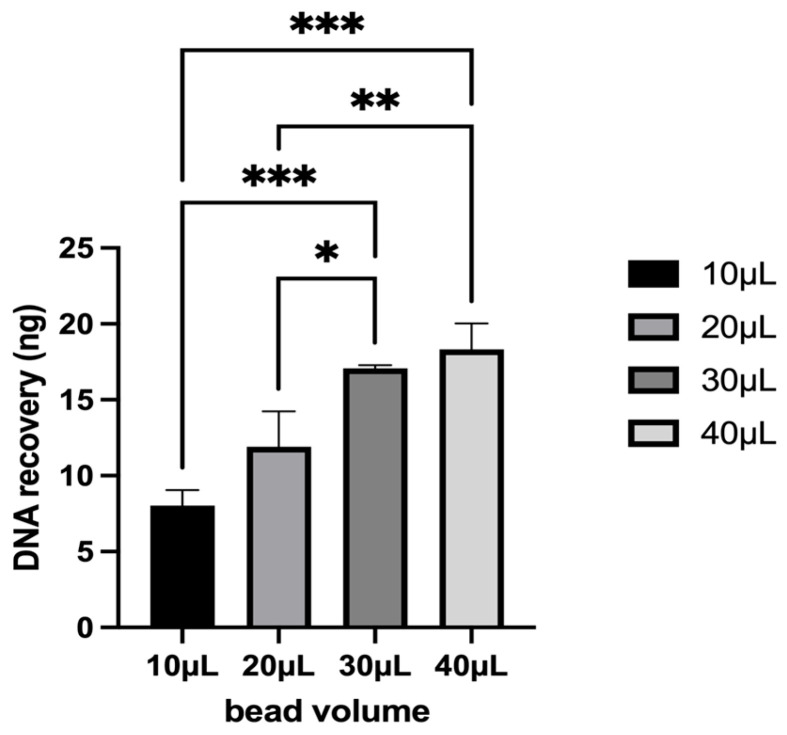
DNA yields obtained from the spiking and re-isolation process for the determination of the optimal magnetic bead volume. Statistical significance was assessed as follows: * *p* < 0.05, ** *p* < 0.01, *** *p* < 0.001.

**Figure A2:**
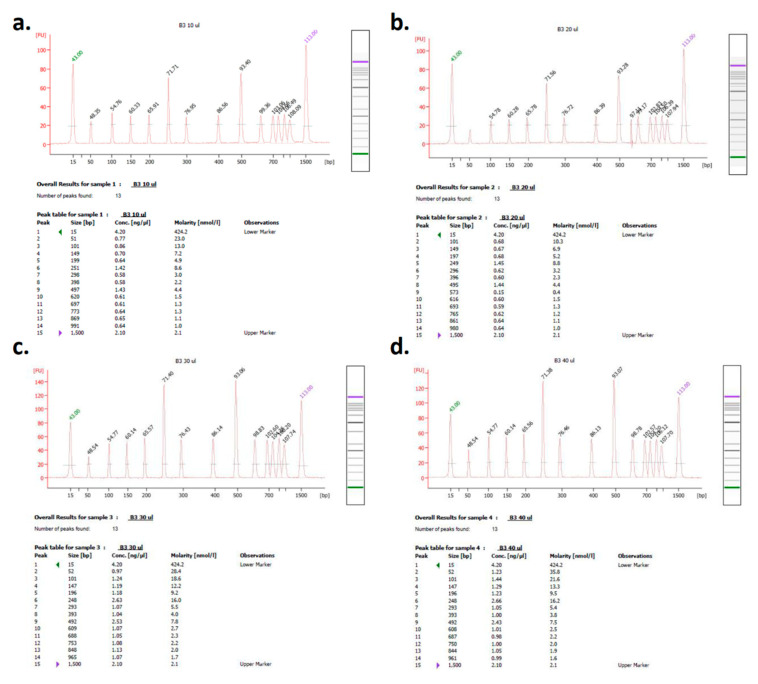
Microelectrophoretic measurements after spiking–re-isolation process for selection of the optimal magnetic bead volumes. (**a**) 10 μL, (**b**) 20 μL, (**c**) 30 μL, and (**d**) 40 μL.

Following the selection of optimal bead chemistry and size, the bead volume was further evaluated. For B4 beads, varying bead volumes (10, 20, 30, and 40 μL) were assessed to determine their efficiency in re-isolation yields. DNA input was 20 ng for each experiment. DNA recovery exhibited a positive correlation with increasing bead volume, as shown in Figure 2. The mean recovery values increased progressively from 10 µL to 40 µL, with 40 µL yielding the highest DNA recovery. A one-way ANOVA was conducted to assess statistical differences among bead volumes, revealing a significant effect. Post-hoc pairwise comparisons indicated that DNA recovery from 30 µL and 40 µL bead volumes was significantly higher than that from 10 µL and 20 µL beads (*p* < 0.01). However, there was no statistically significant difference between 30 µL and 40 µL (*p* > 0.05), suggesting a plateau effect in recovery efficiency beyond 30 µL (Figure 2 and Figure A2).

These findings suggest that increasing bead volume enhances DNA recovery up to 30 µL, after which additional beads do not provide a proportionate increase in yield. This result highlights an optimal bead volume for efficient DNA purification while minimizing reagent consumption. Future optimization should explore recovery efficiency in different sample matrices to validate these observations.

**Figure 3:**
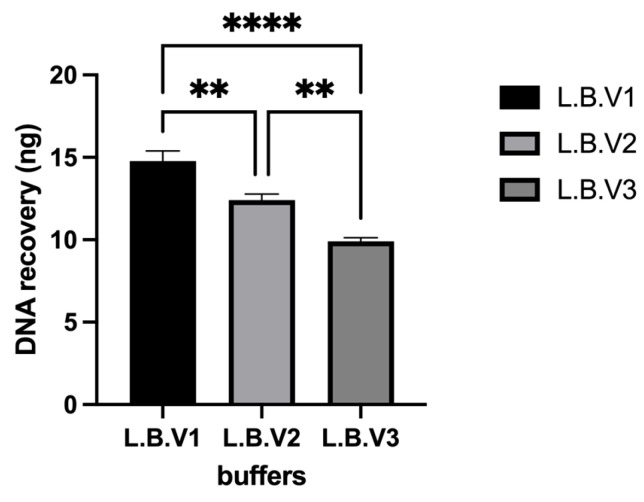
DNA yields obtained from the spiking and re-isolation process for the optimization of lysis buffer composition. LB: lysis buffer; V1–V3: versions 1–3. Statistical significance was assessed as follows: ** *p* < 0.01, **** *p* < 0.0001.

**Figure A3:**
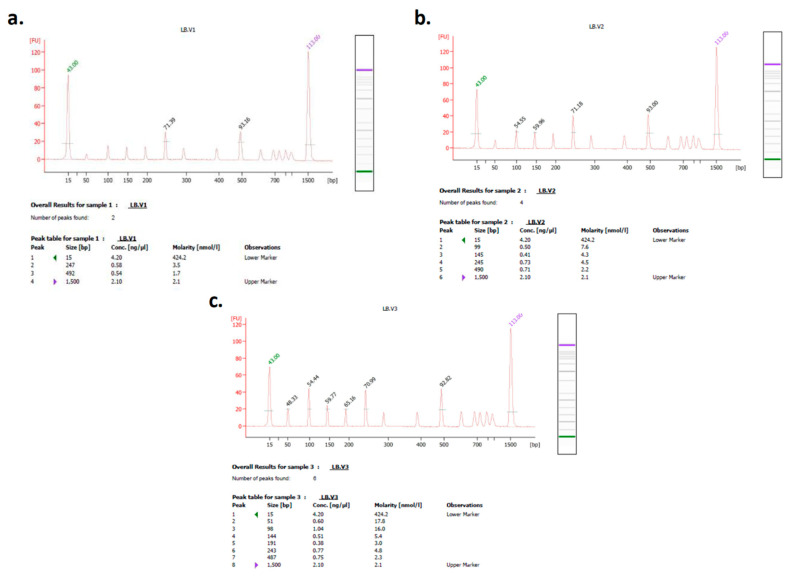
Microelectrophoretic measurements after spiking–re-isolation process to optimize lysis buffer content. (**a**–**c**) Versions 1–3.

In addition to magnetic bead optimization, the volumes and concentrations of other key kit components—including lysis buffer, binding buffer, wash buffers, and proteinase K—were systematically optimized using an oligonucleotide spiking and recovery approach. However, due to commercial considerations, the exact compositions of these components are not disclosed. Nevertheless, their concentrations were systematically adjusted, and isolation yields were evaluated. The ratios of guanidine HCl, nonionic surfactant, Tris base, and capturing agent in the lysis buffer were varied to identify the most effective formulation (Figure 3 and Figure A3).

DNA recovery efficiency varied among the tested buffer formulations (L.B. V1, L.B. V2, and L.B. V3), as shown in Figure 3 and Figure A3. L.B. V1 demonstrated the highest DNA recovery (~16 ng/µL), followed by L.B. V2 (~14 ng/µL), while L.B. V3 exhibited the lowest recovery (~12 ng/µL). A one-way ANOVA indicated a statistically significant effect of buffer type on DNA recovery (*p* < 0.05). Post-hoc multiple comparisons confirmed that L.B. V1 yielded significantly higher DNA recovery than L.B. V2 (*p* < 0.01), and the difference between L.B. V1 and L.B. V2 was also statistically significant (*p* < 0.01). These findings suggest that L.B. V1 is the most effective buffer formulation for maximizing DNA yield, while L.B. V3 demonstrates the lowest efficiency.

### 3.2. Final Protocol

Following the optimization of reagent content, the protocol mentioned in the Materials and Methods was slightly modified to account for plasma volume adjustments. For 1 mL of plasma, the optimized reagent volumes were as follows: proteinase K (20 μL), lysis buffer (50 μL), binding buffer (2.25 mL), and magnetic beads (15 μL). Additionally, the incubation time with the lysis buffer was extended to 20 min to enhance DNA recovery. The final volumes of wash buffers were increased to 800 μL, while the elution buffer volume was reduced to 15 μL for 1 mL plasma samples to optimize DNA yield and concentration.

### 3.3. Limit of Detection Ratio

**Figure 4:**
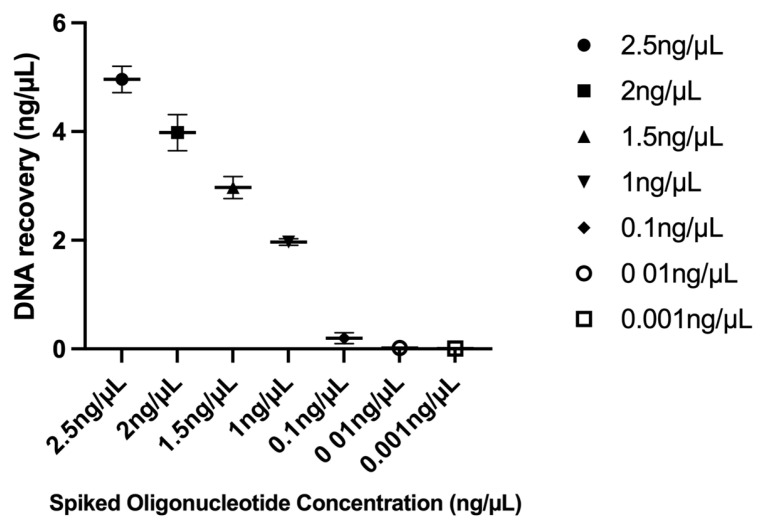
Limit of detection evaluation. DNA recovery obtained from limit of detection studies.

**Figure A4:**
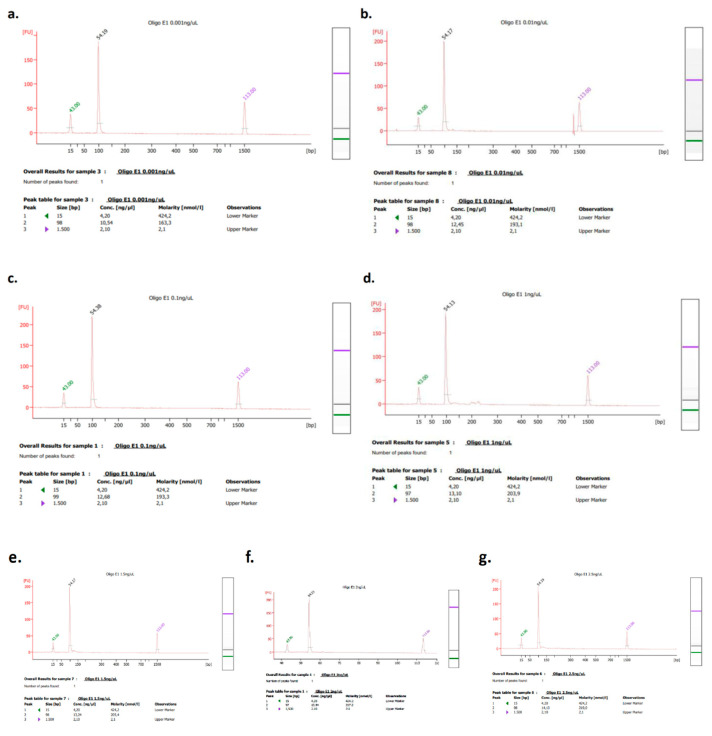
Microelectrophoretic measurements after spiking–re-isolation process for LOD ratios. (**a**–**g**) 2.5–0.001 ng/μL.

The detection limits of the developed kit were assessed by spiking oligonucleotides into human plasma at concentrations ranging from 2.5 to 0.001 ng/μL, with a total spiking volume of 2 μL, corresponding to an absolute quantity of 5 to 0.002 ng of oligonucleotides per sample. As shown in Figure 4 and Figure A4, the kit demonstrated consistent and efficient DNA recovery across the concentration range of 0.1 to 2.5 ng/μL, where recovery remained proportional to input DNA levels, indicating a linear response. Recovery efficiency decreased markedly below 0.1 ng/μL, suggesting that this lower range falls outside the assay’s optimal linear working range. The signal-to-noise ratio (SNR) was calculated by dividing the mean signal by the standard deviation of the background signal. The limit of detection (LoD) was defined as the lowest concentration at which the SNR was ≥3, and the limit of quantification (LoQ) was defined as the concentration at which the SNR was ≥10. Based on these criteria, the LoD and LoQ were determined to be 0.3 pg/μL and 1 pg/μL, respectively. However, since both values fall within the non-linear recovery region (<0.1 ng/μL), their quantitative interpretation should be considered with caution. While the kit can detect and distinguish DNA at these low concentrations, the recovery efficiency is reduced, and results obtained in this range may not reflect the same level of quantitative accuracy observed in the linear range.

### 3.4. Testing PCR Inhibition

**Figure 5:**
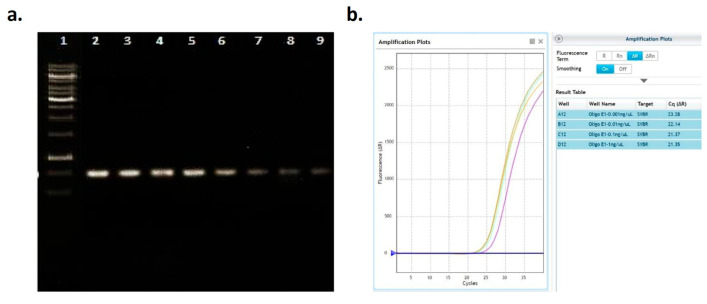
Evaluation of PCR inhibition by developed kit. (**a**) Agarose gel image after standard PCR. 1: 50 bp DNA ladder; 2 and 3: 5 ng; 4 and 5: 4 ng; 6 and 7: 3 ng; and 8 and 9: 2 ng spiked oligonucleotides. (**b**) Amplification curves and cycle threshold (Ct values) obtained by RT-PCR. Oligonucleotide concentrations were 1–0.001 ng/μL.

To assess the potential negative effects of the kit components on PCR, critical for the pre-processing for next-generation sequencing and mutation profiling, plasma-spiked and recovered oligonucleotides were amplified using standard and SYBR Green-based RT-PCR. For both methods (Figure 5), the results showed that the template re-isolated oligonucleotides were efficiently amplified, and no underlying PCR inhibition was detected.

The potential inhibitory effects of the kit components on PCR, a critical step in pre-processing for next-generation sequencing (NGS) and mutation profiling, were assessed by amplifying plasma-spiked and recovered oligonucleotides using both standard PCR and RT-PCR. The results from both methods demonstrated that the re-isolated oligonucleotides were efficiently amplified, indicating the absence of PCR inhibition (Figure 5).

### 3.5. Performance Comparisons

**Figure 6:**
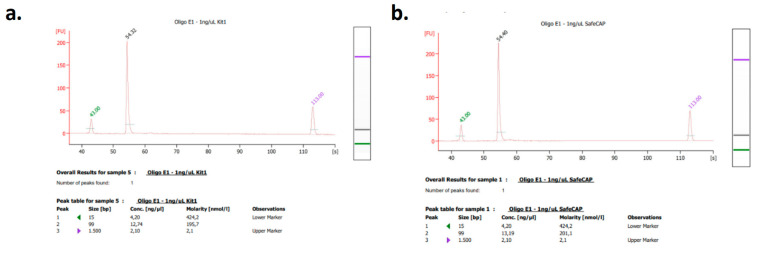
A representative microelectrophoretic measurement result of kit comparisons for 1 ng/μL oligonucleotide spiking and re-isolation with (**a**) the Apostle MiniMax High Efficiency Cell-Free DNA Isolation Kit and (**b**) the SafeCAP 2.0 Cell-Free DNA Extraction and Capturing Kit.

**Figure A5:**
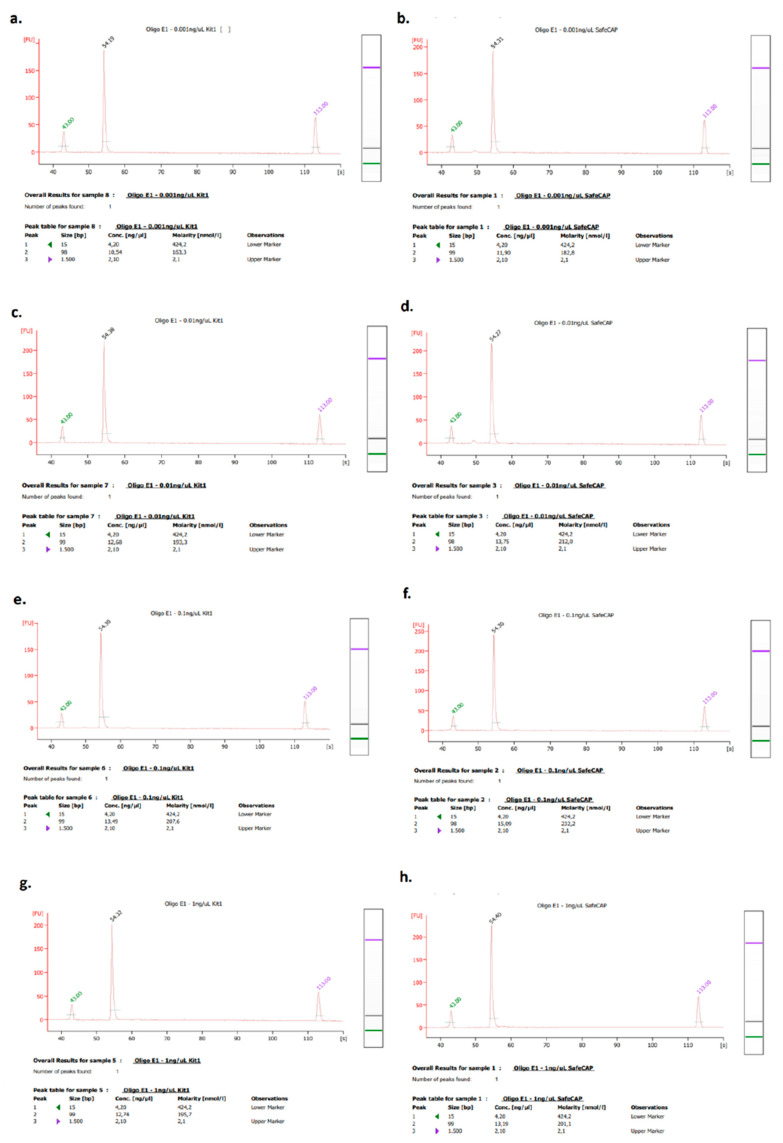
Microelectrophoretic measurements after spiking–re-isolation process for performance comparisons. (**a**) Apostle MiniMax High Efficiency Cell-Free DNA Isolation Kit; (**b**) SafeCAP 2.0 Cell-Free DNA Extraction and Capturing Kit with 0.001 ng/μL spiked oligonucleotide; (**c**) Apostle MiniMax High Efficiency Cell-Free DNA Isolation Kit; (**d**) SafeCAP 2.0 Cell-Free DNA Extraction and Capturing Kit with 0.01 ng/μL spiked oligonucleotide; (**e**) Apostle MiniMax High Efficiency Cell-Free DNA Isolation Kit; (**f**) SafeCAP 2.0 Cell-Free DNA Extraction and Capturing Kit with 0.1 ng/μL spiked oligonucleotide; (**g**) Apostle MiniMax High Efficiency Cell-Free DNA Isolation Kit; and (**h**) SafeCAP 2.0 Cell-Free DNA Extraction and Capturing Kit with 1 ng/μL spiked oligonucleotide.

The performance of the developed kit was evaluated in comparison to a commercially available alternative, the Apostle MiniMax High Efficiency Cell-Free DNA Isolation Kit (Apostle, San Jose, CA, USA), by spiking and recovering oligonucleotides in human plasma. Oligonucleotides at varying concentrations were introduced into plasma samples and subsequently re-isolated using both the SafeCAP 2.0 Cell-Free DNA Extraction and Capturing Kit and the Apostle MiniMax Kit. The recovered oligonucleotides were then analyzed using the 2100 Bioanalyzer System (Agilent Inc., Santa Clara, CA, USA) to assess DNA yield and fragment size. The results demonstrated comparable isolation performance between the two kits in terms of DNA fragment size and concentration (Figure 6 for a representative result; Figure A5 for comparisons across all concentrations).

### 3.6. Clinical Validations

**Figure 7:**
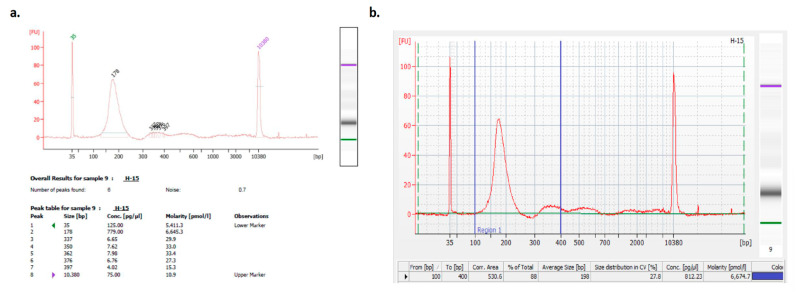
A representative result of microelectrophoretic measurements (**a**) after cfDNA isolation from clinical samples with the developed kit, with electropherogram (**b**). H-15: Patient No. 15.

Following preclinical optimization studies using artificial oligonucleotides, clinical samples from 47 patients diagnosed with stage IV NSCLC were analyzed using the developed kit. The isolated clinical cfDNA was characterized using the 2100 Bioanalyzer System (Agilent Inc., Santa Clara, CA, USA). The results demonstrated high cfDNA isolation yields with no detectable genomic DNA contamination (Figure 7, representative result), thereby confirming the clinical validation of the developed kit.

## 4. Discussion

The findings of this study emphasize the successful development and validation of the SafeCAP 2.0 Cell-Free DNA Extraction and Capturing Kit, demonstrating its robust performance in cfDNA isolation from both spiked plasma and clinical samples, particularly from patients with stage IV non-small cell lung cancer (NSCLC). This novel kit, based on magnetic bead technology, has shown promising results, offering high DNA yield, purity, and reproducibility, which are essential for reliable downstream applications such as PCR and next-generation sequencing.

cfDNA extraction has been shown to be performed by automated systems utilizing magnetic beads, bead-based microfluidics, a liquid-phase extraction strategy using centrifugation, spin columns, and manual magnetic bead-based approaches [35,36,37,38,39,40,41]. Numerous studies have compared the performances of those systems and commercially available kits [42,43,44]. Accordingly, performances of the systems/kits were differently reported in these studies since the isolation of cfDNA could remarkably be affected by preanalytical variables including blood collection tubes, blood storage, and freeze/thaw cycles [45]. Still, recovery rates were documented to be higher for spin column-based kits. However, the ability to capture short fragments was proved to be superior for magnetic bead-based systems [43]. Regarding the instrument requirements and cost of the kit components, the novel SafeCAP 2.0 Cell-Free DNA Extraction and Capturing Kit was designed in a magnetic bead-based approach.

The optimization of various components, particularly the magnetic beads, was crucial in achieving optimal cfDNA isolation. The study systematically tested different bead chemistries and sizes, ultimately identifying the -OH functionalized beads with sizes between 100 and 250 nm as the most effective. This step is significant as it highlights the impact of bead properties on DNA recovery efficiency, particularly for the recovery of fragmented DNA, a critical consideration in cfDNA isolation. The optimization of other kit components such as the lysis buffer, binding buffer, and wash buffers further enhanced the kit’s efficiency, underscoring the importance of each reagent’s concentration and compatibility in achieving high recovery rates.

Importantly, the SafeCAP 2.0 Kit exhibited a limit of detection (LoD) as low as 0.3 pg/µL and showed no PCR inhibition, making it suitable for highly sensitive applications. These performance metrics position it competitively against existing commercial alternatives. Although comparison was limited to the Apostle MiniMax Kit in this study, literature benchmarking suggests that SafeCAP 2.0 performs on par with or better than other kits like the QIAamp Circulating Nucleic Acid Kit, MagMAX, and Maxwell RSC in terms of yield, inhibitor removal, and cost-efficiency [46].

In the context of PCR inhibition, this study showed no detectable inhibition in both standard PCR and RT-PCR, reinforcing the reliability of the extracted cfDNA for subsequent molecular analyses. This is a key factor that differentiates the SafeCAP 2.0 kit from other available commercial kits, ensuring that it does not compromise the accuracy of molecular assays.

When compared with the commercially available Apostle MiniMax High Efficiency Cell-Free DNA Isolation Kit, the SafeCAP 2.0 kit showed comparable performance in terms of DNA yield and quality, as assessed through microelectrophoretic measurements. This is particularly noteworthy because the Apostle kit is widely regarded for its high efficiency. The similarity in performance between the two kits, coupled with the optimized cost and operational simplicity of the SafeCAP 2.0 kit, positions it as a competitive alternative in the field of cfDNA extraction.

In addition to its technical advantages, the SafeCAP 2.0 kit was designed to be cost-effective. The formulation utilizes scalable, non-proprietary raw materials, which significantly reduce production costs. Furthermore, the protocol minimizes hands-on time and the need for consumables, lowering overall per-sample processing expenses. Importantly, the kit is compatible with standard laboratory equipment, eliminating the requirement for specialized instruments or proprietary systems. These features collectively make SafeCAP 2.0 a practical and economical solution for both research and clinical settings, including resource-limited laboratories.

The clinical validation of the SafeCAP 2.0 kit using plasma samples from 47 patients with stage IV NSCLC further supports its applicability in real-world scenarios. NSCLC is a type of cancer in which obtaining a biopsy from tumor tissue is not easily possible, thus cfDNA is critically important to evaluate and manage the carcinogenesis [47]. The high recovery rates of cfDNA from clinical samples without contamination from genomic DNA are essential for ensuring the specificity and accuracy of clinical diagnostics, particularly in cancer genomics. This also emphasizes the kit’s potential utility in routine clinical practice, where the ability to isolate high-quality cfDNA from patient samples is paramount for subsequent molecular analyses, including the detection of genetic mutations and monitoring of therapeutic responses.

From a clinical translation perspective, the kit’s affordability, ease of use, and robust performance in routine lab conditions make it a viable option for widespread adoption, including resource-limited settings. The simplicity of the protocol and minimal equipment requirements support its potential deployment in decentralized laboratories or rural hospitals.

### Study Limitations

While the SafeCAP 2.0 kit demonstrated strong performance in cfDNA recovery and showed comparable results to a leading commercial kit, several limitations of the present study should be acknowledged. First, the comparative performance analysis was conducted against a single commercial reference kit (Apostle MiniMax). Broader benchmarking against multiple established cfDNA extraction platforms, such as the QIAamp Circulating Nucleic Acid Kit, MagMAX, or Maxwell RSC, was not performed and remains an area for future investigation.

Second, while the kit was clinically validated using plasma samples from patients with stage IV non-small cell lung cancer (NSCLC), its applicability to other clinical contexts—including prenatal diagnostics, infectious diseases, or autoimmune disorders—was not evaluated in this study. Expanding validation across a wider range of sample types and pathologies would be necessary to confirm its broader utility. Additionally, the current study focused exclusively on manual workflows. While the protocol is compatible with automation, integration into high-throughput automated platforms was not assessed and should be addressed in future studies to enhance scalability and clinical utility.

Lastly, although the kit demonstrated a limit of detection (LoD) of 0.3 pg/μL and a limit of quantification (LoQ) of 1 pg/μL based on signal-to-noise ratios, both values fall below the assay’s linear recovery range. Consequently, quantitative interpretations at these low concentrations should be made with caution, particularly in applications requiring absolute quantification. Future work should aim to expand clinical testing across diverse sample types and pathologies, assess cross-platform compatibility with NGS workflows, and evaluate the performance of the kit in fully automated extraction systems.

## 5. Conclusions

In conclusion, the SafeCAP 2.0 Cell-Free DNA Extraction and Capturing Kit represents a significant advancement in cfDNA isolation technology. The successful optimization, preclinical validation, and clinical performance of this kit demonstrate its potential for widespread use in both research and clinical diagnostics. By providing a reliable, reproducible, and cost-effective solution for cfDNA extraction, the SafeCAP 2.0 kit holds promise for advancing non-invasive diagnostic tools, particularly in oncology and prenatal testing, as well as other applications involving cell-free DNA analysis.

## Figures and Tables

**Table 1:** Magnetic beads and their chemical properties.

Code	Side Group	Solid Content	Size (nm)
B1	–COOH	3%	400–600
B2			100–300
B3	–OH		350–500
B4			100–250
B5			400–500
B6			140–160

## Data Availability

All data generated or analyzed during this study are included in this published article.
